# Pregnancy associated TMA in 13-year-old patient successfully treated with Eculizumab: case report

**DOI:** 10.1186/s12882-022-02766-y

**Published:** 2022-04-15

**Authors:** Ellen Cody, Donna Claes, Veronica Taylor, Elif Erkan

**Affiliations:** 1 Cincinnati Children’s Hospital Medical Center, Pediatric Nephrology, Cincinnati, OH USA; 2 Department of Pediatric Nephrology, Cincinnati Children’s Hospital Center, University of Cincinnati College of Medicine, Cincinnati, OH USA; 3 Department of pediatric Nephrology, University of Nebraska Medical Center, Children’s Hospital & Medical Center, Omaha, NE USA

**Keywords:** TMA, Pregnancy, Pediatric, Case report, dialysis

## Abstract

**Background:**

This report introduces an unusual cause of kidney failure in a previously healthy pediatric patient. She developed thrombotic microangiopathy (TMA) that was diagnosed post-partum, requiring dialysis and eculizumab, with eventual recovery of kidney function ([chronic kidney disease (CKD) stage 3].

**Case presentation:**

The patient was induced at term due to preeclampsia, with delivery complicated by severe postpartum hemorrhage from uterine atony. She continued to have severe hypertension post-delivery and further developed acute kidney injury (AKI) with decreased urinary output and respiratory distress requiring dialysis therapy. Labs revealed hemolysis with elevated lactate dehydrogenase, low haptoglobin, anemia, and thrombocytopenia, but otherwise unremarkable immunology labs. Once clinically stabilized the patient underwent kidney biopsy, which was consistent with TMA. Treatment was initiated with eculizumab, a monoclonal antibody for terminal complement blockade. Her clinical status improved (including markers of hemolysis and inflammation) with kidney replacement therapy and complement blockade. On discharge, she had increasing urine output and was prescribed 3 day per week hemodialysis and twice monthly eculizumab infusions. By 6 weeks post-delivery, hemodialysis was discontinued and her eculizumab was weaned to monthly infusions. Eculizumab was discontinued at 12 months postpartum. Genetic testing for mutations of the complement system was negative. The patient has residual stage 3 CKD with stable kidney function, requiring two agents for blood pressure control, including an ACE inhibitor for antiproteinuric effect.

**Conclusions:**

This case report showcases an unusual cause of renal failure in a pediatric patient due to TMA in the post-partum period. She required intermittent hemodialysis (iHD) for a brief period, however she was treated successfully with eculizumab that was able to be weaned off 1 year after delivery. She has residual stage 3 CKD and no further signs or symptoms of TMA.

**Supplementary Information:**

The online version contains supplementary material available at 10.1186/s12882-022-02766-y.

## Background

The incidence of pregnancy related acute kidney injury (PR-AKI) has been rising in the United States in the past decade, with recent estimates of the incidence being 2.3 to 4.5 per 10,000 deliveries [1–3]. However, the incidence of pregnancy-associated thrombotic microangiopathy is rare, estimated at 1 in 25,000 pregnancies. The diagnosis can be challenging, as preeclampsia, HELLP syndrome and TMA have overlapping features, and the diagnosis of pregnancy associated HUS is typically one of exclusion. In addition, the role of eculizumab in pregnancy associated TMA is still being explored [4, 5]. This case demonstrates the difficulty of diagnosis and severity of presentation of TMA post-partum, as well as the utility of eculizumab for this disease.

## Case presentation

Patient is a 13-year-old G1P0 female who developed severe acute kidney injury following delivery. Pregnancy was complicated by preeclampsion leading to induction of labor at 37 weeks gestation. At the time of delivery vs Prior to delivery, serum creatine was < 0.5 mg/dl and urinalysis was without proteinuria, although the patient reported headache and malaise. About 3 h post-delivery, she developed severe post-partum hemorrhage secondary to uterine atony, for which she required 3.6 l of blood products. Afterwards, she was noted to have severe hypertension, with blood pressure readings of > 160/90 mmHg. She developed oliguria not responsive to fluid resuscitation or diuretic therapy. Due to developing respiratory distress, she was transferred to the adult medical ICU (MICU) for further management.

### MICU course

On arrival to the ICU, chest imaging showed new infiltrates and the patient was treated for hospital acquired pneumonia. Initial blood cultures grew coagulase negative staphylococcus, treated with vancomycin for 72 h. Her serum creatinine (Cr) on admission to the MICU was 2.24 mg/dL). Given this finding in conjunction with persistent oligo-anuria, a right internal jugular hemodialysis catheter was placed, and iHD was initiated on post-partum day three. Renal ultrasound at that time was normal.

Further lab evaluation revealed proteinuria (urine protein to creatinine ratio 19,000 mg/g), transaminitis (AST 37 unit/L, ALT 229 unit/L), worsening anemia (Hgb dropped from 10.5 to 7.1 g/dL), thrombocytopenia (101 K/mcl nadir, with a recent platelet transfusion given for HD catheter placement), and an elevated LDH (2679 units/L; normal < 325 units/L). She was diagnosed with Hemolysis, Elevated Liver Enzymes, Low Platelets (HELLP) syndrome. She remained persistently hypertensive requiring increasing doses of beta blockers. Due to persistent hypertension and worsening pulmonary edema, she was transferred from the adult hospital to the local pediatric hospital for further management on post-partum day 7.

### Pediatric hospital course

**Fig. 1:**
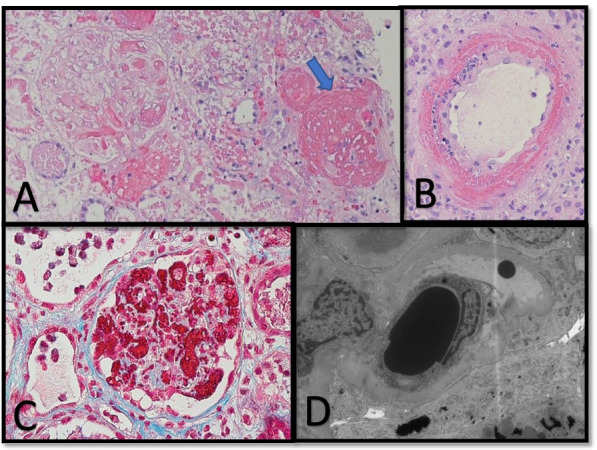
**A** H&E, 400x magnification. Three glomeruli are present, with one glomerulus (arrow) showing complete necrotic injury and others showing thrombi and arteriolar hemorrhage. **B** H&E, 400x magnification. The small arteries show fibrinoid necrosis and endothelial swelling. **C** Trichrome, 400x magnification – Trichrome highlights some of the fibrin thrombi in glomerular capillary loops. **D** Electron Microscopy shows a narrowing of the glomerular capillary lumen with lifting of the plump, reactive endothelium away from the basement membrane and amorphous material between endothelium and BM

**Fig. 2:**
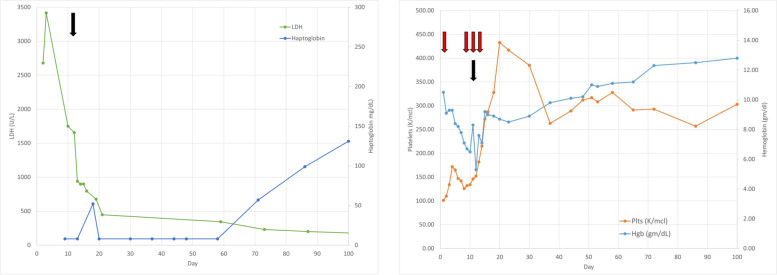
Hemolysis labs during clinical course. Left shows hemoglobin and platelets. Right shows haptoglobin and LDH. Black arrows show first dose of eculizumab

On arrival, the patient had hypertension and respiratory failure requiring BiPAP. Due to fluid overload, she was started on aquapheresis for continuous ultrafiltration. Seven liters of volume was removed in the next 48 h, (Supplementary Fig. 1), with improvement in her blood pressure. Once stabilized, she underwent kidney biopsy on postpartum day 10, which revealed thrombotic microangiopathy (TMA), see Fig. 1. Histology showed thrombi and arteriolar hemorrhage in glomeruli, some with complete necrosis, and small arteries show fibrinoid necrosis and endothelial swelling, with no noted injury to the kidney tubules or interstitium. Additional workup demonstrated normal ADAMTS13 activity (67%) and normal complement evaluation, including sC5B9 (237 ng/mL; normal < 244 ng/mL), C3 (115mg/dL; normal 71–150 mg/dL), C4 (30.2 mg/dL; normal 15.7–47.0 mg/dL), and CH50 (272 units; normal 101–300 units) values, normal factor H and I levels, and absence of Factor H autoantibodies. Hemolysis labs showed elevated LDH at 1751 U/L and low haptoglobin at < 8 mg/dL (See Fig. 2). Shiga toxin testing was not performed due to absence of diarrhea. Liver enzymes were reassuring, with normal ALT at 14 unit/L. The patient was started on complement blockade with eculizumab immediately following the biopsy results (post-partum day 11) after receiving meningococcal immunization (see Supplementary Fig. 2). Penicillin prophylaxis was initiated and continued for duration of eculizumab therapy.

Due to improving volume status, respiratory status, and hematologic parameters, she was rapidly transitioned from continuous renal replacement therapy to iHD (see Supplementary Fig. 1). Soon after eculizumab initiation, her hemolysis improved (see Fig. 2). Her estimated dry weight was eventually established as 62.5 kg (see Supplementary Fig. 1). She received eculizumab 900 mg every 72 h for four doses. Eculizumab efficacy was evaluated by laboratory evaluation of CH50 (goal < 14 U), eculizumab drug levels (goal > 99 mcg/mL), sC5b9 (ng/ml), as well as markers as hemolysis (including hemoglobin and LDH, Fig. 2). On discharge, she was transitioned to maintenance eculizumab dosing of 1200 mg every week. She was undergoing dialysis 3 days per week and began to produce urine.

### Patient follow up

Renal function and urine output continued to improve as an outpatient. CH50 levels and eculizumab levels remained therapeutic (eculizumab 277 mcg/mL, CH50 12 U, Supplementary Fig. 2). Eculizumab dosing frequency was able to be spaced, initially to every 2 weeks followed by every 4 weeks at 6 months after disease presentation, with stability in hemolysis labs (see Fig. 2). By 6 weeks following presentation, she was producing 2 l of urine per day and hemodialysis was discontinued. Her serum Cr stabilized at 1.3–1.5 mg/dL (see Supplementary Fig. 3) with an estimated glomerular filtration rate (GFR) of 53 ml/min/1.73m^2^ and all supplements/therapies for chronic kidney disease were discontinued. She remained only on anti-hypertensive therapy, including an ACE inhibitor for persistent proteinuria (which was initiated once the patient initiated a long-acting contraceptive agent). Her eculizumab was discontinued by 12 months, at which time her markers of hemolysis (LDH, haptoglobin) were normal, with stable platelets and hemoglobin (see Fig. 2). Complement genetic testing panel of 10 genes associated with aHUS (*C3, CFB, CFH, CFHR1, CFHR3, CFHR5, CFI, DGKE, MCP, THBD*) showed no variants of clinical significance.

## Discussion and conclusions

The diagnosis of pregnancy-associated TMA can be difficult, as preeclampsia, HELLP syndrome and pregnancy associated HUS have overlapping features. However, these diagnoses are a spectrum of TMA. Pregnancy related acute kidney injury (PR-AKI) has seen a rising incidence in the United States in the past decade, from 2.3 to 4.5 per 10,000 deliveries, with hypertensive disorders of pregnancy, such as preeclampsia, being a common cause of PR-AKI [1–3]. The incidence of pregnancy-associated TMA is rare, estimated at 1 in 25,000 pregnancies. Pregnancy associated TMA is defined by the occurrence of fibrin and/or platelet thrombi in the microvasculature, leading to mechanical hemolysis and thrombocytopenia during pregnancy or post partum, not resolved by delivery. The only treatment for pre-eclampsia and HELLP syndrome is delivery, with resolution of the associated pre-eclampsia and HELLP syndrome symptoms typically seen within 72 h of delivery [4–6]. Pregnancy-associated HUS in pregnancy is associated with dysregulation of the alternative complement pathway and is often a diagnosis of exclusion [4, 5]. Pregnancy-associated HUS primarily occurs postpartum in three-fourths of cases, and the combination of LDH > 600 U/L with Cr > 1.9 mg/dL has been found to be associated with differentiating pregnancy-associated HUS from HELLP in the postpartum period [4–7]. Our patient fits this description, with post-partum onset, elevated LDH and Cr, as well as the pathologic presentation of fibrin and platelet thrombi in the microvasculature. The persistent hemolysis with progressive renal failure and hypertension (which persisted beyond 72 h post-partum), as well as the exclusion of TTP, also support the diagnosis. TMA is uncommon among patients presenting with malignant hypertension caused by diseases other than pregnancy-associated HUS, making malignant hypertension an unlikely cause of the patient’s disease [8].

Risk factors for P-HUS involve dysregulation of the complement system, preeclampsia, postpartum hemorrhage, placental abruption, nulliparity and personal or family history of atypical HUS [1]. A large proportion of patients with pregnancy-associated HUS can have severe AKI requiring dialysis; long-term complications can include chronic kidney disease versus kidney failure that must be treated with either chronic dialysis therapy and/or an eventual kidney transplant [1, 2, 5, 6]. Historically, treatment for pregnancy-associated HUS included plasma exchange, with response rate around 50% [5]. In the era of complement blockade with medications such as eculizumab, outcomes have greatly improved [9]. No controlled clinical trials have been performed; however, it is estimated that nearly 90% of patients achieve hematologic and renal remission. Long term outcomes and duration of treatment remains unclear, and a repeat pregnancy would require multidisciplinary assessment as it would be high-risk both for mother and baby [5]. A recent study from Rondeau et al [10] evaluated 44 pregnancies in 41 patients with a previous diagnosis of aHUS, of which 24 pregnancies were exposed to eculizumab. There were four patients that each reported 1 TMA event during pregnancy, with only 1 receiving eculizumab prior to the TMA event. The subsequent patients went on to receive eculizumab, two of whom went on to deliver healthy babies.

This case presents an example of a pediatric patient without a known complement gene abnormality. Given her age, rapid deterioration, kidney biopsy findings and lack of modifiable inciting factors, she was started on eculizumab, even with otherwise normal complement labs. In addition, she had persistent findings of hemolysis on labs, with elevated LDH and low hemoglobin, requiring continued blood transfusions. She was treated with standard induction and maintenance therapy of eculizumab through the first 6 months. On initiation of eculizumab, her hemoglobin stabilized, and she did not require further blood transfusion. Due to clinical stability, as well as complement and hemolysis lab stability, eculizumab was successfully able to be weaned and discontinued with continued remission, both by hematological and renal parameters. She has had no further evidence of HUS in the 2 years since her initial presentation. She did develop stage 3 CKD, however kidney function has remained stable despite discontinuation of eculizumab without relapse of HUS.

## Supplementary Information

**Additional file 1: Supplementary Figure 1.** Weight over admission. Green arrows represent HD initiation, each dot representing weight post-treatment. Red line represents time on aquapheresis. Purple line represents time on CRRT. Following transition off CRRT back onto HD, patient’s last HD treatment is marked by orange arrow.

**Additional file 2: Supplementary Figure 2.** Blue points represent patient eculizumab levels throughout disease course. Dashed blue line is target eculizumab level (99 mcg/mL). Green points represent patient’s CH50 level throughout disease course, with dashed green line representing goal CH50 suppression (12 U). Red/blue points represent patient’s sC5b9 levels through course, with dashed red line representing goal level (244 ng/mL). Black arrow represents first and last eculizumab doses.

**Additional file 3: Supplementary Figure 3.** Serum Creatinine over clinical course. Green arrow represents HD initiation and final treatment.

## Data Availability

Not applicable.

## Abbreviations

TMA: Thrombotic Microangiopathy
CKD: Chronic Kidney Disease
AKI: Acute kidney injury
PR-AKI: Pregnancy related acute kidney injury
CR: Creatinine
HELLP: Hemolysis, Elevated Liver Enzymes, Low Platelets
iHD: Intermittent hemodialysis
GFR: Glomerular filtration rate
HUS: Hemolytic Uremic Syndrome
MICU: Medical Intensive Care Unit
