# Supplementary figures and images for: Pregnancy associated TMA in 13-year-old patient successfully treated with Eculizumab: case report
Source: BMC Nephrol. 2022 Apr 15;23:147. doi: 10.1186/s12882-022-02766-y (PMC9013145; doi:10.1186/s12882-022-02766-y)

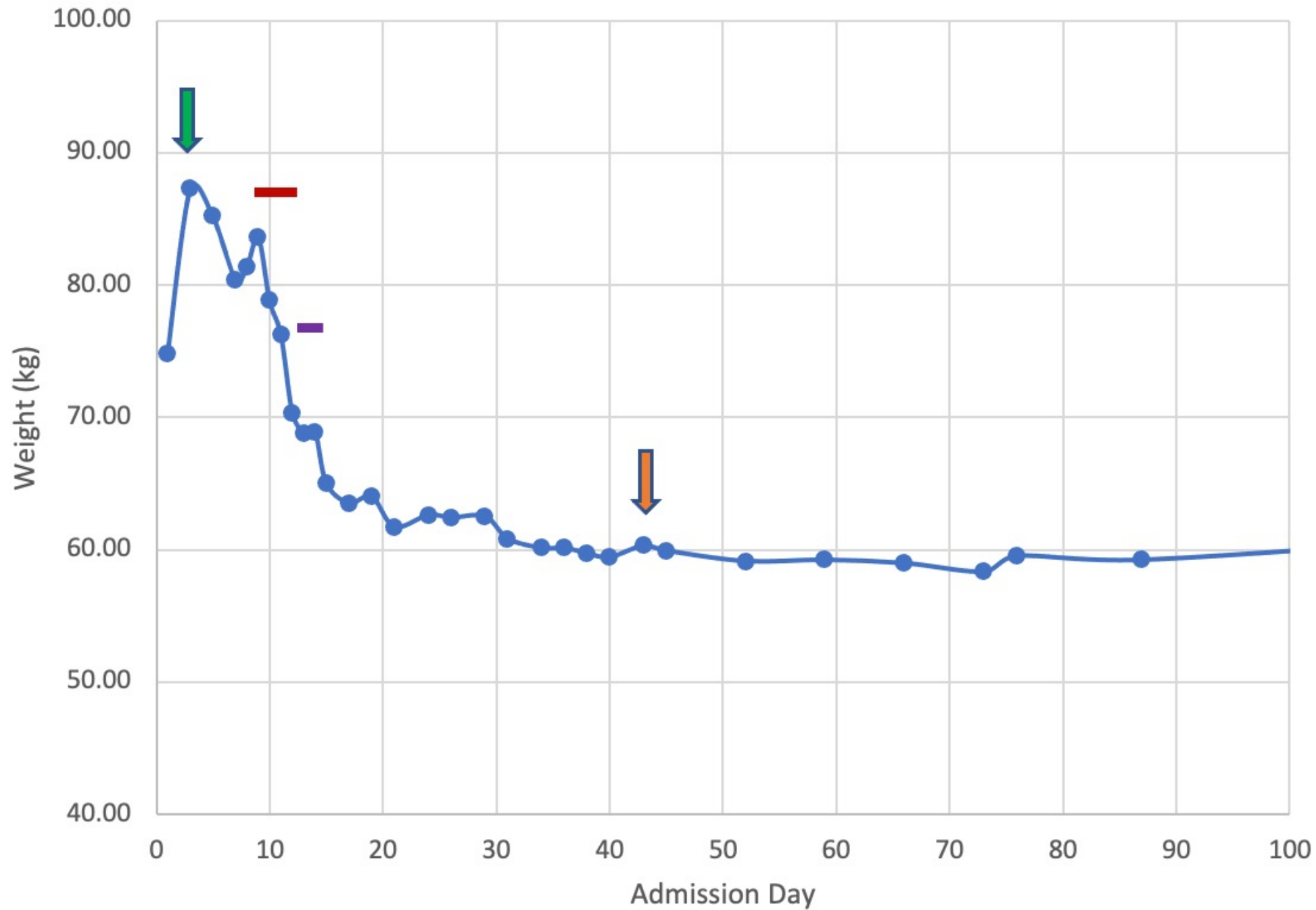

Supplement: Supplementary file 1 — Additional file 1: Supplementary Figure 1. Weight over admission. Green arrows represent HD initiation, each dot representing weight post-treatment. Red line represents time on aquapheresis. Purple line represents time on CRRT. Following transition off CRRT back onto HD, patient’s last HD treatment is marked by orange arrow. [file 12882_2022_2766_MOESM1_ESM.pdf]

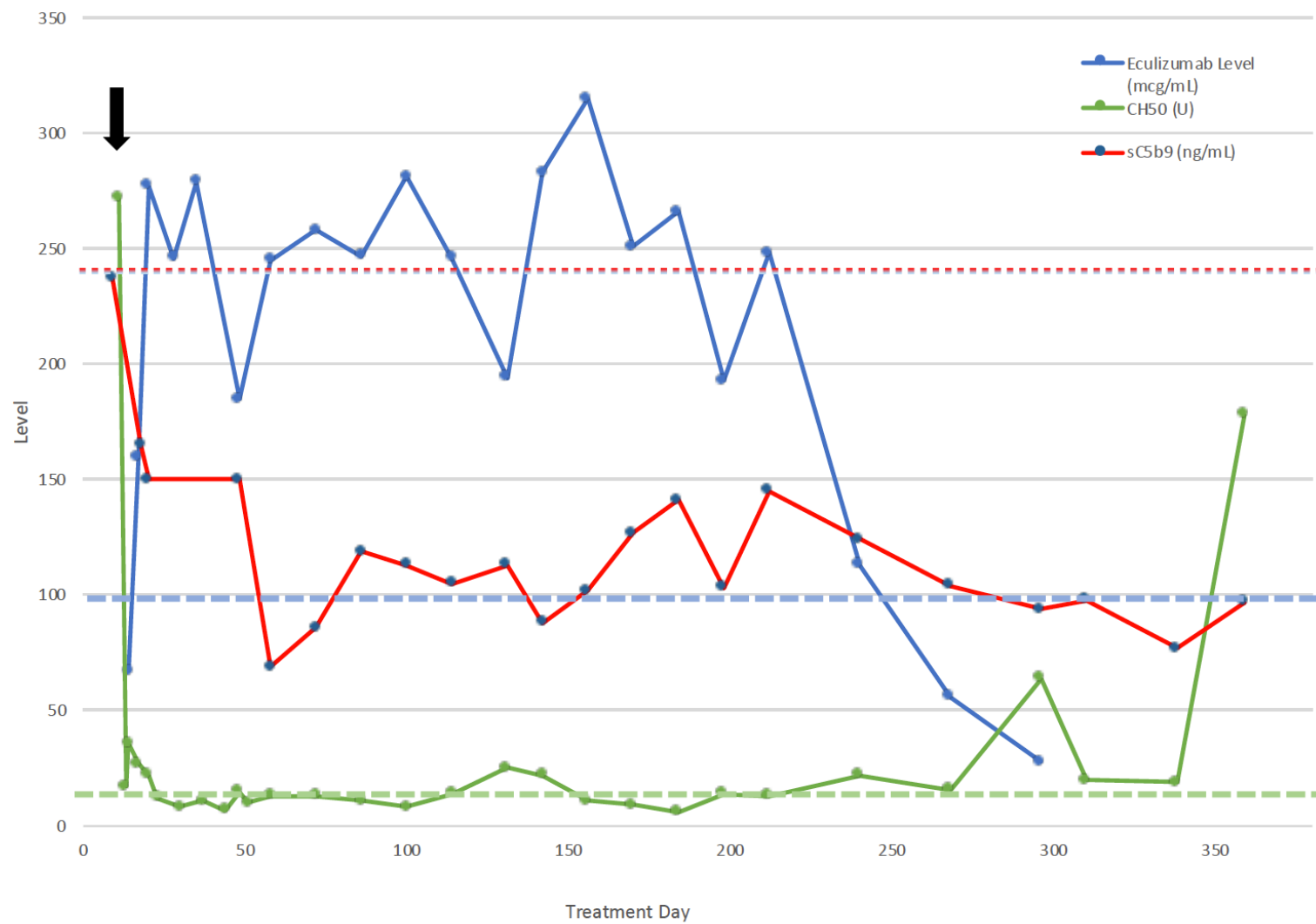

Supplement: Supplementary file 2 — Additional file 2: Supplementary Figure 2. Blue points represent patient eculizumab levels throughout disease course. Dashed blue line is target eculizumab level (99 mcg/mL). Green points represent patient’s CH50 level throughout disease course, with dashed green line representing goal CH50 suppression (12 U). Red/blue points represent patient’s sC5b9 levels through course, with dashed red line representing goal level (244 ng/mL). Black arrow represents first and last eculizumab doses. [file 12882_2022_2766_MOESM2_ESM.pdf]

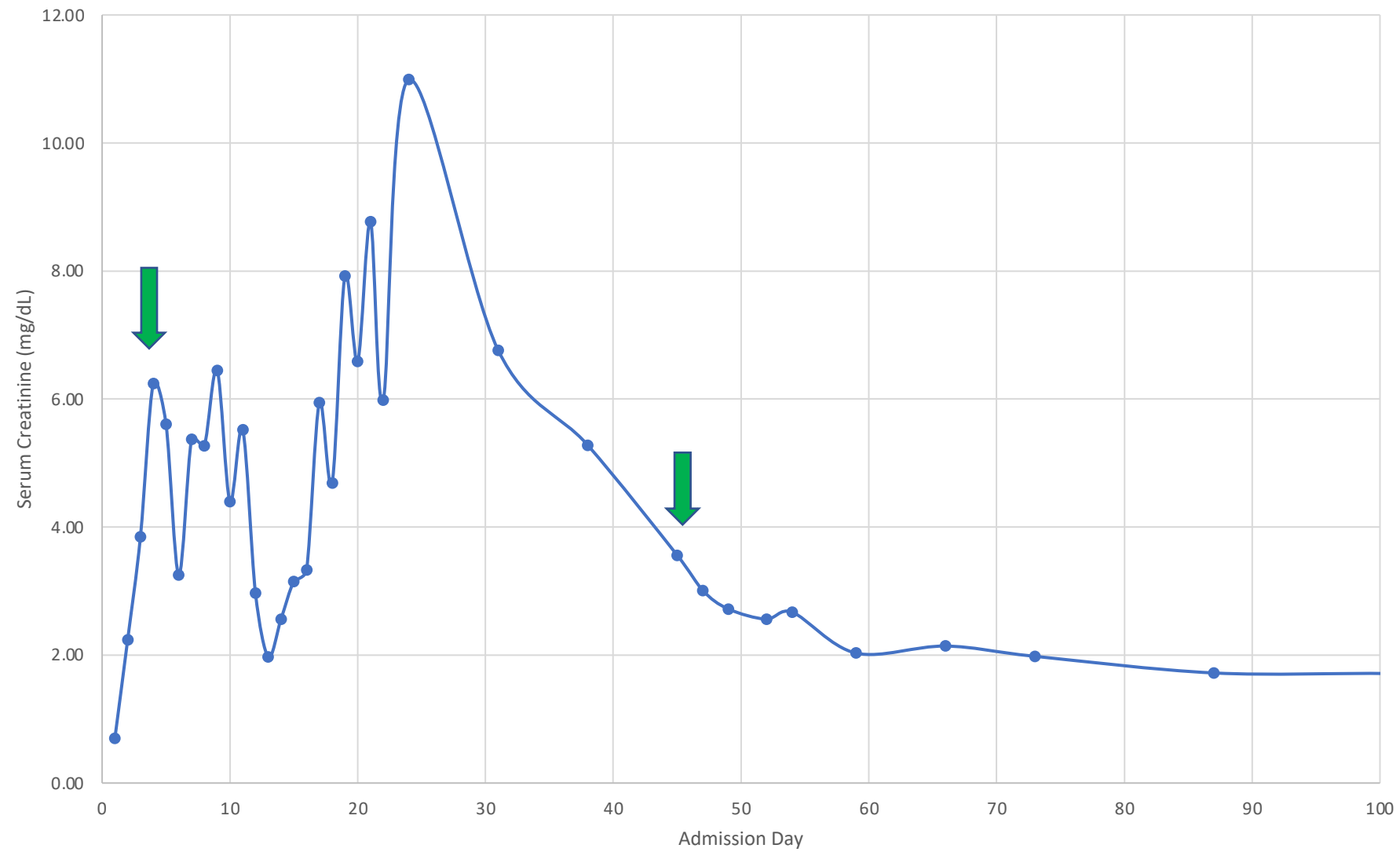

Supplement: Supplementary file 3 — Additional file 3: Supplementary Figure 3. Serum Creatinine over clinical course. Green arrow represents HD initiation and final treatment. [file 12882_2022_2766_MOESM3_ESM.pdf]
